# Gene therapy and peripheral nerve repair: a perspective

**DOI:** 10.3389/fnmol.2015.00032

**Published:** 2015-07-15

**Authors:** Stefan A. Hoyng, Fred de Winter, Martijn R. Tannemaat, Bas Blits, Martijn J. A. Malessy, Joost Verhaagen

**Affiliations:** ^1^Department of Neuroregeneration, Netherlands Institute for NeuroscienceAmsterdam, Netherlands; ^2^Department of Neurosurgery, Leiden University Medical CenterLeiden, Netherlands; ^3^Department of Neurology, Leiden University Medical CenterLeiden, Netherlands; ^4^UniQureAmsterdam, Netherlands; ^5^Center for Neurogenomics and Cognition Research, Neuroscience Campus AmsterdamAmsterdam, Netherlands

**Keywords:** gene therapy, adeno-associated viral vector, lentiviral vector, neurosurgery, Schwann cell

## Abstract

Clinical phase I/II studies have demonstrated the safety of gene therapy for a variety of central nervous system disorders, including Canavan’s, Parkinson’s (PD) and Alzheimer’s disease (AD), retinal diseases and pain. The majority of gene therapy studies in the CNS have used adeno-associated viral vectors (AAV) and the first AAV-based therapeutic, a vector encoding lipoprotein lipase, is now marketed in Europe under the name Glybera. These remarkable advances may become relevant to translational research on gene therapy to promote peripheral nervous system (PNS) repair. This short review first summarizes the results of gene therapy in animal models for peripheral nerve repair. Secondly, we identify key areas of future research in the domain of PNS-gene therapy. Finally, a perspective is provided on the path to clinical translation of PNS-gene therapy for traumatic nerve injuries. In the latter section we discuss the route and mode of delivery of the vector to human patients, the efficacy and safety of the vector, and the choice of the patient population for a first possible proof-of-concept clinical study.

## Gene Therapy in Animal Models for PNS Injury

**Figure 1:**
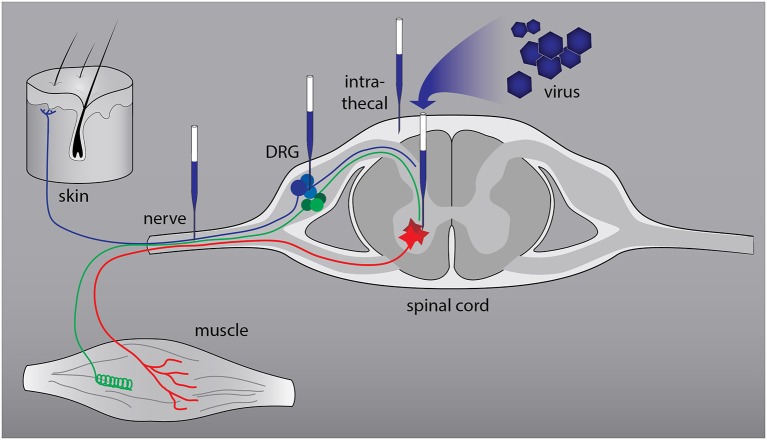
**Anatomical relationships in the peripheral nervous system (PNS) and sites of viral vector-mediated gene delivery.** The PNS consists of primary sensory neurons (blue and green: nociceptive and proprioceptive neurons) in the dorsal root ganglia (DRG) and motor neurons (red) in the ventral horn of the spinal cord. The axons form a mixed nerve that innervates the skin and muscle. Successful gene delivery to primary sensory and motor neurons and to Schwann cells, the resident glia cells of peripheral nerves, has been reported with various viral vectors. To target primary sensory and motor neurons two routes of delivery have been used successfully: direct intraganglionic or intraspinal injection and intrathecal (IT) delivery. Injection of a viral vector in the nerve stump distal to the lesion or in a nerve graft that bridges the lesion results in transduction of Schwann cells.

The peripheral nervous system (PNS) consists of primary sensory neurons in the dorsal root ganglia and motor neurons in the ventral horn of the spinal cord (Figure 1). Most peripheral nerves contain axons of sensory and motor neurons and patients who sustain an injury experience loss of sensory and motor function. In patients regeneration of injured peripheral axons does occur but is almost never complete. This is due to the low velocity of axon growth, the deterioration of pro-regenerative Schwann cells in the distal nerve stump following longer periods of denervation, and the misrouting of regrowing axons (Brushart, 2011; Allodi et al., 2012). Nerve regeneration is studied in well-defined rodent models of nerve injury. A widely used model is transection of the sciatic nerve of the rat followed by end-to-end repair of the nerve stumps or implantation of an autograft or artificial nerve guide to bridge the gap between the stumps. In this model axons reinnervate the end organs within weeks to months. Cervical or lumbar spinal root avulsions followed by reimplantation of the roots are much more severe injuries (Eggers et al., 2010; Chu et al., 2012). Following cervical lesions it can take up to 12 weeks before the first axons reinnervate target cells, whereas a significant proportion of axons will stall in the nerve and never reach the end organ. In the lumbar root avulsion model functional recovery is minimal and it is therefore one of the best possible mimics of chronic denervation in human patients with proximal lesions.

Surgical repair of peripheral nerves has reached its optimal refinement. Recovery of function as a result of surgical repair has significantly improved but remains limited. Novel adjuvant therapeutic strategies to promote axon regeneration in the injured peripheral nerve are needed to further improve recovery of function. One of these strategies is gene therapy. Successful gene delivery to primary sensory and motor neurons and to Schwann cells, the resident glia cells of peripheral nerves, has been reported with various viral vectors (Haastert-Talini, 2011; Mason et al., 2011). Herpes simplex viral vectors attracted early interest because of their natural tropism for sensory neurons (Geller and Breakefield, 1988; Glorioso and Fink, 2009). Adeno-associated viral vectors (AAV) vectors have become popular as gene delivery agents for neurons of the PNS for several reasons. AAV have a low risk of insertional mutagenesis and immunogenicity, they lack endogenous viral genes, can be produced at high titer and at clinical grade (Salmon et al., 2014; Felberbaum, 2015; Hastie and Samulski, 2015). There are at least 12 vector serotypes, and a number of AAV variants engineered by e.g., viral evolution, which display distinct transduction profiles (Kotterman and Schaffer, 2014). AAV5 is the serotype of choice for rat sensory neurons (Mason et al., 2010), whereas AAV2, 6, 9 and rh10 efficiently target spinal motor neurons (Peel et al., 1997; Blits et al., 2004; Snyder et al., 2011; Homs et al., 2014; Hordeaux et al., 2015). AAV vectors have been used to study the effects of a variety of genes on regeneration of the central branch of sensory neurons (Andrews et al., 2009; Bareyre et al., 2011; Parikh et al., 2011) and on the survival of motor neurons (Blits et al., 2004; Homs et al., 2014; Pajenda et al., 2014; Hordeaux et al., 2015).

Schwann cells are central to the success of peripheral nerve regeneration. However, the unique pro-regenerative properties of these cells fade away after longer periods of denervation. Most gene therapy studies used lentiviral vectors to promote the therapeutic potential of Schwann cells transplanted in artificial nerve guides or in nerve sheets (Haastert et al., 2006; Li et al., 2006; Shakhbazau et al., 2012; Godinho et al., 2013; Santosa et al., 2013) of Schwann cells in autografts (Hoyng et al., 2014a), of Schwann cells present in damaged nerves distal to an injury (Tannemaat et al., 2008; Esaki et al., 2011) or in spinal roots reimplanted in the spinal cord (Eggers et al., 2008). Increased expression of neurotrophic factors is one of the key events observed following peripheral nerve injury. Neurotrophic factor gene therapy stimulated axon regeneration (Mason et al., 2011), myelination (Haastert et al., 2006, 2008; Homs et al., 2011) and facilitated the return of compound motor action potentials (Allodi et al., 2014). Moreover, nerve growth factor (NGF)-gene therapy was used to promote directional growth of sensory axons (Hu et al., 2010). Unexpectedly, however, persistent expression of NGF or glial cell line-derived neurotrophic factor (GDNF) did cause excessive, modality specific axon growth and trapping at the site of expression thereby prohibiting distal growth of axons toward the skin or muscle (Tannemaat et al., 2008; Santosa et al., 2013; Hoyng et al., 2014a). On the one hand, these observations highlight the unprecedented potency of neurotrophic factors. On the other hand they underscore the need to control the dose and timing of these therapeutic proteins. In the next section three key future areas of research will be discussed, including the optimization of the transduction of Schwann cells, development of gene switches to control the timing of transgene expression, and the need to better understand the biology of the pro-regenerative properties of Schwann cells.

## Key Areas of Future Research

**Figure 2:**
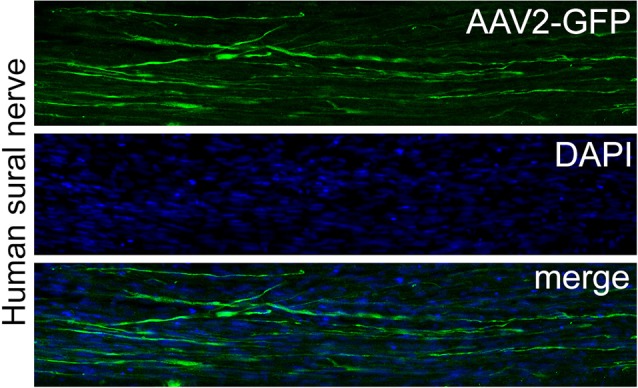
**AAV2-mediated transduction of a human sural nerve segment.** Surplus human nerve material was obtained from the operation room and anonymized as stated in the code of conduct for responsible use of human tissue and medical research (Federa, 2011). Nine AAV serotypes were compared for their transduction efficiency by injecting 1.85 × 10^10^ gc/cm nerve and culturing the nerve segments for 14 days. After 14 days nerve segments were immersion fixed with paraformaldehyde and 20 μm sections were prepared. The upper panel show a section through a human sural nerve stained for GFP. The middle panel shows the same image stained for the nuclear stain Hoechst and the lower panel shows the merged images of the two panels. AAV2 transduced numerous cells that display the typical longitudinal shape of Schwann cells and this serotype was superior to all other serotypes tested. More details on this study can be found in Hoyng et al. (2015).

AAV is gaining increasing acceptance as a clinical gene delivery platform (Hastie and Samulski, 2015). However, in animal studies PNS-gene therapy to enhance the performance of Schwann cells largely relied on lentiviral vector or adenoviral vector-mediated gene delivery (Mason et al., 2011), with the exception of one recent study that used AAV (Homs et al., 2011). Lentiviral vectors integrate their genetic information into the host cell genome, whereas transgene expression via adenoviral vectors rapidly declines as a result of immune-mediated toxicity (Hermens and Verhaagen, 1997; Dijkhuizen et al., 1998). Although the overall risk of lentiviral vector-associated insertional mutagenesis is low (De Palma et al., 2005; Montini et al., 2006), lentiviral vectors could potentially be harmful for the transduced cells. Surprisingly, very little information is available on the transduction of Schwann cells with AAV vectors (Homs et al., 2011). A recent comparative study of nine AAV serotypes and lentiviral vectors shows that optimal transduction of rat and human Schwann cells is achieved by different serotypes. Rat nerve segments could be genetically modified equally well by a set of four AAV vectors (AAV1, 5, 7, 9), whereas AAV2 was superior in human nerve segments (Hoyng et al., 2015; Figure 2). Transduction with lentiviral vectors was, however, superior to the best AAV vectors. Thus, a *first key area of future research* would be to further optimize gene delivery to Schwann cells, either by identifying newly engineered AAV vectors with an improved tropism for Schwann cells (Kotterman and Schaffer, 2014), or by testing lentiviral vectors with an improved safety profile, e.g., non-integrating lentiviral vectors (Yáñez-Muñoz et al., 2006; Cesana et al., 2014). *In vivo* electroporation of expression plasmids in Schwann cells could be an alternative to viral vector-directed gene delivery (Aspalter et al., 2009; Pereira Lopes et al., 2013). Plasmid-mediated gene transfer is a straight forward procedure, however, the strong electrical currents required for the electroporation, the relatively low transduction rate, and short-lived expression of the therapeutic gene indicate that* in vivo* plasmid-based gene transfer will have limited utility.

A *second area of key future research* concerns the creation of a safe regulatable gene therapy vector. In the context of PNS-gene therapy this is essential for two reasons. First, persistent expression of certain growth factors leads to local trapping of axons (discussed above). Second, continued growth factor expression may have unacceptable side-effects, e.g., Nerve growth factor (NGF) may induce hypersensitivity (Verge et al., 2014). The criteria for regulated vector-based therapeutic gene expression are that: (1) it can be induced by a small molecule that is safe; (2) it can be turned off effectively by withdrawal of the inducer whereas “leaky” expression should be minimal and preferably undetectable; and (3) the transactivator protein (TA) that is employed should be non-immunogenic and well-tolerated. The prototypical system for regulating gene expression involves a TA that binds to a promoter in the presence of doxycycline. However, the TA is a bacterial protein and is therefore a permanent immunological target (Markusic and Seppen, 2010). Clinical use of analogous systems using alternative TAs is precluded for the same reason. Viruses have evolved several strategies to escape immune surveillance (Zaldumbide and Hoeben, 2008). We took advantage of the long Gly-Ala repeat (GAr) domain of Epstein Barr virus (Yin et al., 2003) to generate an immunologically inert version of the TA. This idea was based on the observation that Schwann cells which express a foreign protein (e.g., green fluorescent protein, GFP) are cleared from the nerve by an immune response. This does not happen when these proteins are fused to GAr (Ossevoort et al., 2006; Hendriks et al., 2007). We fused GAr to TA and showed that GAr-TA retains its sensitivity to doxorubicin (dox). This system has been used to turn GDNF expression “on” and “off” in rat sciatic nerve. The GAr-TA system is several fold less “leaky” compared to the TA protein. GAr-TA displays strongly reduced immunogenicity in a bioassay for antigenic peptide generation. Therefore, the GAr-TA system fulfills many of the criteria for safe regulatable gene expression (Hoyng et al., 2014b). However, GAr-TA requires doxycycline concentrations that are 40-fold higher than clinically acceptable levels. Therefore, current studies focus on testing newer versions of the TA (Das et al., 2008) and shorter GAr tags which may have improved doxycycline sensitivity. Another complication that may occur is that the continuous presence of the immune-inert TA in cells may induce unwanted effects. Therefore efforts are ongoing to regulate therapeutic and TA gene expression simultaneously. Apart from ligand (i.e., doxcycyline) regulated promoters, promoters induced by physiological stimuli associated with neural injury may emerge as tools to restrict transgene expression to the post-lesion period (Jazwa et al., 2013). The glia fibrillary acidic protein (GFAP)-promoter is an example of an injury induced promotor that has been used in transgenic mice to turn on gene expression in a diseased peripheral nerve (Keller et al., 2009). However GFAP continues to be expressed in non-myelinating Schwann cells in an intact nerve which would result in some level of persistent transgene expression after nerve regeneration has been completed.

Optimization of gene delivery to Schwann cells and the creation of safe regulatable gene therapy vectors are biotechnological challenges. A *third area of future research* concerns the gathering of fundamental biological know-how on the cellular and molecular properties of Schwann cells in a regenerating nerve. A nerve injury induces major, tightly coordinated changes in gene expression in Schwann cells in the distal nerve. Together with the typical alignment of Schwann cells in pathways for growing axons, this creates a unique environment for successful regeneration. The signals that transform stable Schwann cells into the specialized repair cells in an injured nerve are not clearly understood and it is not known why Schwann cells gradually lose their pro-regenerative properties after longer times of denervation (Gordon et al., 2011). Moreover, growing evidence indicates the existence of Schwann cells with distinct phenotypes preferentially supporting either motor or sensory neuron regeneration (Wright et al., 2014) which is relevant to direct growing axons to their correct target cells.

To develop new strategies to stimulate axon regeneration, an analysis of the mechanisms that underlie the pro-regenerative properties of Schwann cells is needed. Conditional knock-out of the gene for the transcription factor c-Jun in Schwann cells has a negative impact on axon regeneration and results in simultaneous down-regulation of multiple pro-regenerative proteins in Schwann cells in an injured nerve (Arthur-Farraj et al., 2012). Neurotrophic factor expression in Schwann cells can be enhanced by overexpression of c-Jun (Huang et al., 2015). C-Jun appears to be one, of perhaps a small set, of central transcriptional “master switches” which, in a cooperative manner, control the pro-regenerative phenotype of Schwann cells (Hung et al., 2015). If, in future experiments, the key regulatory complex of transcription factors are identified, these genes would be prime targets for Schwann cell gene therapy. “Transcriptional reprogramming” of Schwann cells is fundamentally different from PNS-gene therapy with a vector encoding a single neurotrophic factor because this would result in an elaborate repertoire of molecular changes (Huang et al., 2015), which would be particularly beneficial during the intermediate and later phases of the regeneration process when the ability of Schwann cells to support axonal outgrowth deteriorates (Gordon et al., 2011). The identification of the transcriptional “master switches” and studies on their combinatorial role in determining the repair-properties of Schwann cells may also shed new light on the occurrence of specific “motor” and “sensory” specific Schwann cells (Wright et al., 2014).

## Path to a Clinical Study

The preclinical issues discussed above require several more years of systematic research in rodents. A clinical study to promote PNS regeneration by gene therapy is therefore currently hypothetical. However, the rapidly growing clinical experience with gene therapy for other neurological diseases and the steady advances in preclinical PNS-gene therapy support the conception of a framework for a future clinical study. The development of a PNS-gene therapy study will benefit particularly from experience with gene therapy for pain and neuromuscular diseases. In these disorders the sensory (pain) and motor neurons and muscle cells (neuromuscular disorders) are the primary target cells (Pleticha et al., 2014a; Cheever et al., 2015). Gene therapy for traumatic nerve injury has to include methods for safe gene transfer to the nerve Schwann cells as well. The following three topics need careful consideration in the context of preclinical-to-clinical translation of PNS-gene therapy and will be discussed below: (1) the route and mode of delivery of the vector; (2) the efficacy and safety of the vector; and (3) the choice of the patient population.

### Route and Mode of Delivery of the Vector

Pleticha and colleagues presented a roadmap for the preclinical evaluation of AAV-based genetic modification of dorsal root ganglia (DRG) for clinical trials on pain (Pleticha et al., 2014a). This roadmap covers the essential preclinical steps needed to realize safe AAV-mediated targeting of primary sensory neurons in human patients. The human DRG is approximately 50 times larger than the rat DRG (Shen et al., 2006). The rat motor neuron pool that supplies the nerves that innervate the forepaw (equivalent to the brachial plexus in humans) spreads over 0.5 cm of cervical cord, whereas the motor neuron pool innervating the brachial plexus in humans spans at least 10 cm of the spinal cord. The longest rat peripheral nerve, the sciatic nerve, is approximately 12 cm long while the nerves that innervate the human arm measure 80– 100 cm. Therefore, translating gene therapy to the PNS of humans poses specific challenges with respect to the route and mode of delivery of the vector because of the diverging anatomical dimensions of the rodent and human PNS (Pleticha et al., 2014a).

To target primary sensory and motor neurons two routes of delivery have successfully been used: direct intraganglionic or intraspinal injection and intrathecal (IT) delivery. In the rat a single intraganglionic injection of an AAV vector results in efficient transduction of sensory neurons with very little if any spread of the vector to other locations (Mason et al., 2010). In contrast, IT delivery results in transduction of sensory and spinal motor neurons and other non-neuronal cell types (Snyder et al., 2011). In humans, lumbar puncture is a relatively safe and standard technique to approach the cerebrospinal fluid and it would be feasible to deliver a vector to human DRGs and spinal motor neurons via this route. AAV vectors were delivered to the cat, the pig and to non-human primates using a lumbar puncture technique (Bucher et al., 2013; Gray et al., 2013; Pleticha et al., 2013; Samaranch et al., 2013; Dirren et al., 2014; Passini et al., 2014). If expression of a transgene in areas outside the DRG is not desirable, direct injection would be a requirement. Convection enhanced delivery (CED) relies on enhanced extracellular transport of a solution infused in tissue over an extended period of time (typically ranging from 20 min to 2 h, Krauze et al., 2005a, b) and results in equal tissue distribution of the infusate. Minimally invasive intra-ganglionic gene transfer by CT-guided percutaneous injection and CED of AAV1 in lumbar DRGs of the pig resulted in 33% transduction of DRG neurons (Pleticha et al., 2014c).

Gene transfer to the injury-repair site of a human peripheral nerve will require a method to deliver a vector to a sural nerve graft inserted to connect the proximal and distal stump or to the nerve distal to the repair site. In rats, when relying on diffusion of the viral vector during a single manually guided 1– 2 μl injection, the vector spreads in a nerve graft or in a nerve stump distal to a repair site over several millimeters (Tannemaat et al., 2008; Hoyng et al., 2014a). Four injections placed at 5– 8 mm distances from each other resulted in the transduction of a 4– 5 cm long segment of rat sciatic nerve (Eggers et al., 2013). This injection technique results in rather unequal transduction of Schwann cells, with “hot spots” containing many transduced cells, and areas with no or very little transduced cells. CED carries macromolecules (such as Gadolinium-labeled Albumine for direct monitoring of the infusion process) over a distance of 1 cm in a rat nerve (Pleticha et al., 2014b) and over distances of 2.7– 3.5 cm in a nerve of a non-human primate (Ratliff and Oldfield, 2001; Chen et al., 2011). Importantly, and in contrast to manual injection of small volumes of vector solution, CED resulted in an equal distribution of the infusate over the nerve. Future studies have to test whether CED of a viral vector to an injured nerve of a larger animal is a feasible option. Taken together, gene therapy for traumatic nerve injuries will benefit significantly from the encouraging observations in larger animals which show that the neuroanatomical dimensions do not preclude efficient gene delivery to the human PNS.

### Safety and Efficacy of the Vector

Rigorous toxicity, and serological and cellular immune assessments have been performed for AAV1, AAV2, AAV5 AAV8 and AAVrh10. These serotypes have been used in clinical trials for lipoprotein lipase deficiency (LPLD; AAV-1; Scott, 2015), Canavan disease, PD and AD (AAV-2; Leone et al., 2000, 2012; Kaplitt et al., 2007; Richardson et al., 2011; Bartus et al., 2013; Rafii et al., 2014), liver mediated diseases (AAV5; Grosios and Pañeda, 2013), San Fillipo B (AAV5, AAVrh10; Tardieu et al., 2014)^1^ and Hemeophilia B (AAV-5, AAV-8; Nathwani et al., 2014). Although most humans have natural occurring neutralizing antibodies against AAV and treatment with AAV usually results in enhanced levels of these antibodies, this occurred without detectable pathological effects (Salmon et al., 2014). Screening of patients following application of an AAV-1 vector to skeletal muscle resulted in seropositivity for AAV1 (Ferreira et al., 2014; Salmon et al., 2014). Antibodies which develop after the administration of AAV1 would not interfere with the therapeutic effect as the AAV vector has already delivered its therapeutic cargo. However, preexisting antibodies may interfere significantly with the transduction process as has been shown in some studies (Samaranch et al., 2013), whereas neutralizing antibodies had no effect on gene delivery with AAV after intraparenchymal or IT injection in other studies (Gray et al., 2013).

Transduction differences between different serotypes in rat, larger animals and human complicates the choice of the vector for preclinical-to-clinical translation. The use of primary human tissue, either biopsy material or autopsy tissue, may prove to be critical in determining the optimal serotype for human patients. In our hands, cultured human peripheral nerve segments, obtained as left-over tissue from the operation theater after nerve repair surgery, were transducible by lentiviral vectors (Tannemaat et al., 2007), whereas AAV-serotype testing showed that AAV2 was superior to eight other common serotypes investigated (Hoyng et al., 2015; Figure 2). To date, AAV2 has been used in several clinical trials and, together with AAV1, is one of the best characterized serotypes. AAV2 outperforms other serotypes in human nerve segments and is therefore currently the leading vector for a clinical study that aims at enhancing the therapeutic potential of Schwann cells in a human peripheral nerve.

### The Choice of the Patient Population

Animal models will provide information about the efficacy and safety of the delivery technique, the vector and the transgene. However, the predictive value of animal studies is limited and eventually a study on a small number of human subjects with a PNS-lesion will be a necessary step in the translation process (Cheever et al., 2015). An early gene therapy study for AD enrolled eight patients (Tuszynski et al., 2005). This study was too small to demonstrate efficacy, but showed that the gene therapy procedure was feasible and well-tolerated. The transgene was NGF, a growth factor relevant in the context of PNS-gene therapy. NGF expression was detectable in post-mortem brain tissue of a subject that died of causes unrelated to the gene delivery procedure. This shows that a small clinical study can be highly informative and may form the basis of a larger randomized gene therapy trial (Cheever et al., 2015).

Nerve injury is a heterogeneous condition, ranging from brachial plexus injuries to distal injuries of the digital nerves that innervate the hand. Established guidelines on the design of clinical trials for the evaluation of novel treatments for nerve injury do not (yet) exist. Previous trials to test experimental treatments to promote nerve regeneration involved patients that sustained very different types of injuries. A recent successful clinical trial on the beneficial effect of electrical stimulation was performed on patients with complete transection injury of the digital nerve (Wong et al., 2015). An advantage of this study population is its relative homogeneity. Although a clinically meaningful degree of regeneration occurs spontaneously in these patients, enhanced sensory reinnervation was detectable following a short period of per-operative electrical stimulation. A follow-up trial with electrical stimulation as adjuvant treatment to surgical repair in patients with a severe brachial plexus injury, a severe lesion that causes serious dysfunction of the arm with prospects of only limited functional recovery of biceps function, is currently underway.^2^ Thus, although electrical stimulation is a straight-forward procedure shown to be effective and safe in animals (Al-Majed et al., 2000; Brushart et al., 2005; Gordon et al., 2008, 2010; Haastert-Talini et al., 2011), tolerability and efficacy were first studied in a patient population that sustained a lesion with relatively moderate medical consequences before translating the procedure to lesions associated with long-lasting disability. A similarly cautious and phased translational path for PNS-gene therapy is mandatory.

Gene therapy for neurotrophic factors was well-tolerated in Alzheimer’s (NGF; Rafii et al., 2014) and Parkinson’s disease (Neurturin; Bartus et al., 2013) patients. In contrast to neurotrophic factor gene therapy in the brain, neurotrophic factor gene delivery to an injured peripheral nerve is not without risk, as it may induce uncontrolled growth of axons, hypersensitivity and unwanted changes in Schwann cells (Mason et al., 2011). As discussed above, animal studies must first provide robust experimental evidence showing that control over the dose and the timing of viral vector-derived neurotrophic factor expression is effective, before gene therapy in a small group of patients with a nerve lesion can be undertaken. A gene therapy study in patients with a digital nerve injury, as performed for electrical stimulation, may reveal potential unwanted effects, and monitoring benefit is possible with the current battery of sensory tests (Wong et al., 2015). The vector, preferably an immune-inert regulatable AAV vector encoding NGF (a growth factor with stimulatory effects on sensory fibers) would be delivered to the denervated digital nerve by CED or by multiple injections along the 6– 10 cm long digital nerve immediately following end-to-end repair. While this pilot study could be important in demonstrating safety and tolerability of PNS gene therapy, the therapeutic benefit of gene therapy for digital nerve injury patients is probably limited as this nerve displays a significant degree of spontaneous regeneration. Patients with a brachial plexus injury, a lesion which has a permanent negative impact, are a target group where gene therapeutic intervention could develop into a genuine adjuvant regenerative treatment strategy to further promote repair after neurosurgical intervention.

## Conflict of Interest Statement

The authors declare that the research was conducted in the absence of any commercial or financial relationships that could be construed as a potential conflict of interest.

## Footnotes

^1^http://www.uniqure.com/news/182/182/Clinical-trial-launched-to-treat-Sanfilippo-B-syndrome-using-gene-therapy.html

^2^www.clinicaltrials.gov
